# Delay in diagnosis and treatment among adult multidrug resistant tuberculosis patients in Yangon Regional Tuberculosis Center, Myanmar: a cross-sectional study

**DOI:** 10.1186/s12913-018-3715-4

**Published:** 2018-11-20

**Authors:** Ye Minn Htun, Tin Mi Mi Khaing, Yin Yin, Zaw Myint, Si Thu Aung, Tin Maung Hlaing, Ngamphol Soonthornworasiri, Udomsak Silachamroon, Yuthichai Kasetjaroen, Jaranit Kaewkungwal

**Affiliations:** 1 Department of Tropical Hygiene, Faculty of Tropical Medicine, Mahidol University, Bangkok, Thailand; 2 Regional Tuberculosis Center, Yangon, Myanmar; 3 National Tuberculosis Programme, Department of Public Health, Ministry of Health and Sports, Nay Pyi Taw, Myanmar; 4 Defence Services Medical Research Centre, Nay Pyi Taw, Myanmar; 5 Department of Clinical Tropical Medicine, Faculty of Tropical Medicine, Mahidol University, Bangkok, Thailand; 6 Bureau of Tuberculosis, Bangkok, Thailand

**Keywords:** Delay, Diagnosis, Treatment, Multidrug-resistant tuberculosis, Myanmar

## Abstract

**Background:**

Delays in diagnosis and treatment initiation may allow the emergence of new cases by transmission to the community, and is one of the challenges facing programme management of drug resistance in Myanmar. This study aimed to explore delays in diagnosis and treatment initiation, and associated factors among patients with multidrug-resistant tuberculosis.

**Methods:**

A cross-sectional study was conducted at Yangon Regional Tuberculosis Centre, Myanmar. Data were collected by face-to-face interviews and treatment-card reviews of all adult patients who had registered and started treatment with the standard regimen from May to November, 2017. Delay time was categorized by using median cut-off and analyzed using SPSS version 23.0. Logistic regression analysis was performed to assess the relative impact of predictor variables on diagnosis and treatment delays.

**Results:**

A total of 210 patients participated in this study. The median diagnosis delay was 9 days, IQR 3 (8–11) and 58.6% of the patients experienced a long diagnosis delay. Below middle school education (adjusted odds ratio [AOR] = 2.75, 95% CI = 1.22–6.21), non-permanent salaried employment (AOR = 3.03, 95% CI = 1.32–6.95), co-existing diabetes mellitus (AOR = 5.06, 95% CI = 1.97–13.01) and poor awareness (AOR = 2.99, 95% CI = 1.29–6.92) were independent predictors of long diagnosis delay. The median treatment delay was 13 days, IQR 9 (8–17) and 51% of the patients experienced long treatment delay**.** Age 31–50 years (AOR = 4.50, 95% CI = 1.47–13.97) and age > 50 years (AOR = 9.40, 95% CI = 2.55–34.83), history with MDR-TB patient (AOR = 3.16, 95% CI = 1.29–7.69), > 20 km away from a Regional TB Centre (AOR = 14.33, 95% CI = 1.91–107.64) and poor awareness (AOR = 4.62, 95% CI = 1.56–13.67) were independent predictors of long treatment delay.

**Conclusions:**

Strengthening comprehensive health education, enhancing treatment adherence counseling, providing more Xpert MTB/RIF machines, expanding decentralized MDR-TB treatment centers, ensuring timely sputum transportation, provision of a patient support package immediately after confirmation, and strengthening contact-tracing for all household contacts with MDR-TB patients and active tuberculosis screening were the most effective ways to shorten delays in MDR-TB diagnosis and treatment initiation.

## Background

Multidrug-resistant tuberculosis (MDR-TB) is a major challenge facing tuberculosis (TB) control programs and a leading public health concern in many countries. MDR-TB is TB with resistance to at least two main drugs—isoniazid and rifampicin [1]. Globally, in 2016, there were an estimated 600,000 incident cases of MDR-TB including rifampicin resistance (RR), with an estimated 4.1% being new cases and 19% being previously treated cases [2]. Early diagnosis and treatment are crucial to programmatic management in countries with a high MDR-TB burden. Delay in the treatment initiation allows transmission to others and new MDR-TB cases emerging in community [3].

Roll-out of new rapid diagnostic method, Xpert MTB/RIF, was designed to reduce delays in treatment process through improved accessibility and faster laboratory turnaround [4]. The World Health Organization (WHO) recommended Xpert MTB/RIF test, a cartridge-based fully automated nucleic acid amplification test, for patients suspected to have MDR-TB and those with HIV-associated TB [5–7]. It can improve both MDR/RR-TB and drug-sensitive TB case detection. Although scaling up of diagnostic capacity and patient-centered care could provide to improve linkage of MDR diagnosis to initiation of treatment, a particular problem in low- and middle-income countries was waiting lists of confirmed MDR-TB patients who were waiting health system capacity to deliver treatment [4].

Myanmar, one of 30 highest MDR-TB burden countries worldwide listed by WHO*,* had 13,000 incident cases of MDR/RR-TB and incidence rate was 25 per 100,000 population in 2016 [2]. The National Tuberculosis Programme (NTP) has implemented programmatic management of drug-resistant TB (PMDT) since 2009. Following WHO’s 2010 policy recommendation, three Xpert MTB/RIF machine were introduced in 2011. The NTP expanded 49 Xpert MTB/RIF machines for improving diagnostic capacities and another six machines through partners by the end of 2015 [8, 9]. Xpert MTB/RIF is a very specific diagnostic test used in point-of-care settings to confirm the patients with RR [10–14] and the RR specimen can be considered as a surrogate marker for MDR-TB [8, 15, 16]. Therefore, LPA and Liquid culture were decided not to include in MDR-TB diagnosis algorithm and Xpert MTB/RIF was recommended as an initial diagnosis test of MDR-TB confirmation in 2017 [8]. All of RR-positive patients tested by Xpert MTB/RIF were enrolled in MDR-TB programme and treated with standard regimen.

Despite on-going efforts to fight MDR-TB, most patients in high-burden countries were experiencing delays in treatment initiation of months or even years because of health care system failures [17–19]. Delays in diagnosis and treatment also result from social discrimination, stigma and a lack of awareness regardless of enhancements in diagnosis and treatment facilities [20, 21]. There were 108 MDR-TB project townships and the NTP was functioning with 42 centers for MDR-TB treatment and care by the end of 2016 [22]. However, the patients with geographical barriers and socioeconomic burden cannot access early diagnosis and treatment and continue to experience delay in health care services [17, 23]. Although the numbers being enrolled on treatment (1497 cohorts in 2014) have doubled over the past year (667 cohorts in 2013) after revising the diagnostic algorithms with optimizing usage of Xpert MTB/RIF, treatment scale-up has not kept pace with diagnosis. Among 17 Regional/States TB Centers, Yangon Region had the highest number of MDR-TB cases (1364) treated with second-line anti-TB drugs during 2016 [22]. The National Strategic Plan for TB intended to enroll the treatment to all MDR-TB patients within two weeks of their diagnosis in 2020 [9]. There is no previously published study conducted in Myanmar to explore delays in diagnosis and treatment among adult MDR-TB patients. The main objectives of this study were to explore delays in diagnosis and treatment initiation, and associated factors among adult MDR-TB patients.

## Methods

### Study setting

A cross-sectional descriptive study was conducted among patients with MDR-TB who were started treatment in Yangon Regional TB Center from May to November 2017. The Xpert MTB/RIF was used for diagnosis of drug susceptible TB as well as initial diagnosis of MDR/RR-TB to eliminate waiting list and to access early treatment initiation [8, 15, 24]. Xpert MTB/RIF was performed for the diagnosis of MDR-TB in patients with risk factor for resistance, diagnosis of TB/MDR-TB in HIV-positive patients, diagnosis of TB in HIV-negative patients with no significant risk for MDR-TB, and diagnosis of TB/MDR-TB from extra-pulmonary specimen in patients with risk factor for TB.

**Fig. 1:**
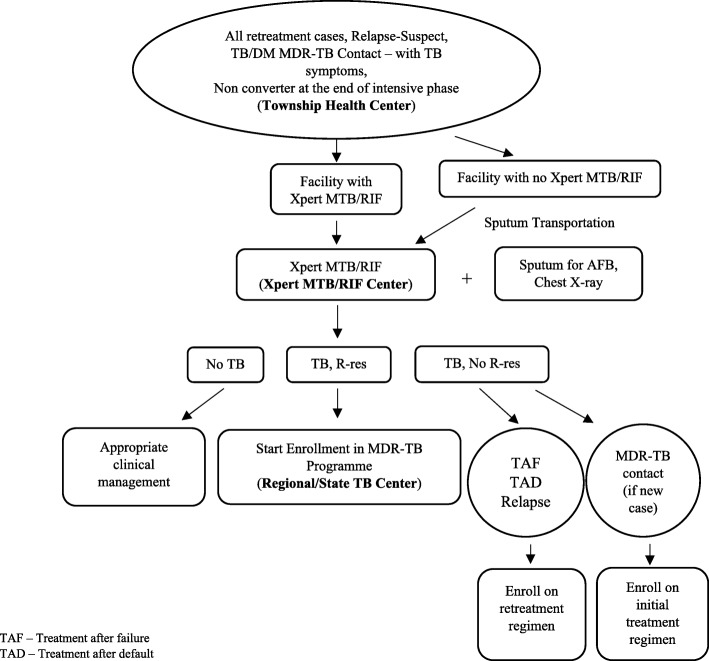
Diagnosis of MDR-TB in patients with risk factor for resistance

The patients categorized to be tested for MDR-TB were referred to the Xpert MTB/RIF Center for confirmation after registration at the Township Health Center. The diagnosis algorithm for the patients at high risk for resistance was shown in Fig. 1. For the result of no TB in HIV-positive patients**,** liquid culture and drug susceptibility testing (DST) or line probe assay (LPA) were done again in National TB Reference Laboratory if they were strongly suspected of TB/MDR-TB. The diagnosis of MDR-TB suspected non-risk patients were needed to perform with two Xpert MTB/RIF tests [8, 24]. Confirmed results were conveyed by phone and posted to the Township Medical Officer, who informed the confirmed result to the patient through Basic Health Staff, and then referred the patient to the Regional TB Center for treatment initiation [8]. Baseline investigations (complete blood count, liver function tests, renal function tests, thyroid function tests, blood sugar, serum electrolytes, pregnancy testing) were conducted at the date of registration at Regional TB Centre and treatment was started after getting results.

All of registered MDR-TB patients were enrolled on standardized treatment regimen which was 20 months of total treatment length and it might be extended depending on the sputum conversion [8, 15]. The intensive phase was 6–8 months with injection amikacin, pyrazinamide, levofloxacin, ethionamide, cycloserine, and 12–14 months of continuous phase using levofloxacin, ethionamide, cycloserine and pyrazinamide. Each dose was given under directly observed treatment short-course (DOTS) throughout the treatment, performing either facility-based or home-based at their respective Township Health Center. They were followed up monthly at Regional TB Center for treatment monitoring. In addition to receiving free drugs and follow-up investigation services, patients were provided with US$30 per month and nutritional support, through funding mainly from the Global Fund and other co-partner organizations, to prevent non-adherence [8, 15].

### Study population and sampling

As there has been no published data in Myanmar regarding delays in diagnosis and treatment among adult MDR-TB patients, the estimation was thus based on a previous study done on proportion of patients with diagnosis and treatment delays in China which showed 37%; with the 95% confident interval and margin of error of 7%, the sample size was calculated at 182, and with adjustment for possibility of incomplete data 210 patients were recruited into the study [25, 26]. In this study, 210 patients with MDR-TB who were registered and started enrollment within study period (May to November 2017) were included. Patients aged younger than 18 years old, severely ill patients and those who were unwilling to participate in the interview were excluded.

### Definition of variables

**Fig. 2:**
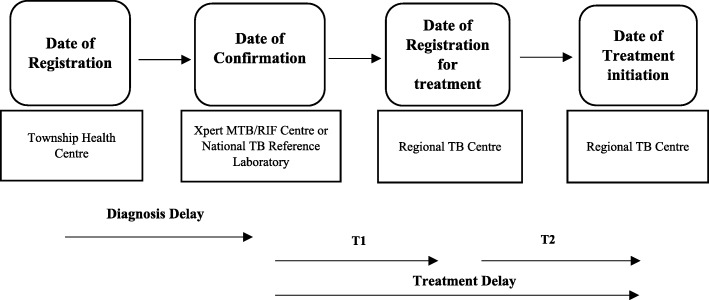
Definition of diagnosis delay and treatment delay used in this study

The definitions of patient categories were in line with drug resistant TB management guideline of NTP which was followed by the WHO definitions [1, 8]. As shown in Fig. 2, diagnosis delay was defined as the period from the date of registration at the Township Health Center until the date of MDR-TB confirmation. Treatment delay was defined as the period from the date of confirmation until the date of treatment initiation [27, 28]. Treatment delay was divided into two parts, T1 and T2, based on registration date at Regional TB Centre to identify the time lasting of baseline investigations that were taken before treatment initiation. T1 was the period started from the date of MDR-TB confirmation and continued until the date of registration at the Regional TB Center. T2 was the period started from the date of registration until the date of treatment initiation at the Regional TB Center. For HIV-negative TB patients with no significant MDR-TB risk, date of diagnosis by second Xpert MTB/RIF test was used as the date of confirmation. Severely ill patient was defined as the one who could not respond to interview due to severe comorbidities, persistent coughing with breathlessness, and weakness with extreme weight loss.

### Data collection

To identify factors associated with delays, the structured questionnaire was constructed based on “Guidelines for the Management of Multidrug-Resistant Tuberculosis (MDR-TB) in Myanmar, 2013” [15] and previous literatures. The questionnaire constituted four parts including patients’ characteristics, level of knowledge, awareness and stigma related MDR-TB.

After the patients received an explanation of the purposes of the study and provided informed consent, the investigator and two trained interviewers collected information from the participants by face to face interview using pre-tested structured questionnaire. To assure the quality of the data, the dates of registration at Township Health Center, confirmation as MDR-TB, registration at Regional TB Centre and treatment initiation were reviewed from MDR-TB treatment cards and medical records of participants. The diagnosis and treatment delay were expressed in days.

### Data management and analysis

The collected data were entered into Excel spreadsheet version 2016, and exported to statistical package for the social sciences (SPSS) software version 23.0 for analysis. Descriptive statistics were presented as number (percentages) for categorical variables and median (Interquartile range, IQR) or mean (Standard deviation, SD) for continuous variables. The normality of different delay periods was checked using Shapiro-Wilk test and all delay periods were not normally distributed. Kaplan-Meier curves provided information about different types of delay. Logistic regression analysis was performed to assess the relative impact of the predictor variables on the outcome variables. Univariate analysis was performed on each independent variable and respective crude odds ratio (COR) was calculated. In order to control of confounders, multivariate analysis was performed with all of variable from univariate analysis to detect the independent predictors of long diagnosis delay and long treatment delay. The significant association of independent variables with dependent variable was assessed by using 95% confidence intervals (95% CI) and respective adjusted odds ratio (AOR). A two tailed-sided *p*-value < 0.05 was considered statistically significant.

### Ethical considerations

The study was approved by the Ethics Committee, Faculty of Tropical Medicine, Mahidol University, Thailand, and the Institutional Review Board, Defence Services Medical Research Centre, Myanmar. Permission for data collection and data review from the Yangon Regional TB Centre was obtained from the NTP, Department of Public Health, Ministry of Health and Sports, Myanmar.

## Results

### Characteristics of patients with MDR-TB

A total of 210 adult patients who were registered and started enrollment in the MDR-TB treatment regimen were selected. As shown in Tables 1, 62.4% were men and 37.6% were women. The mean ± standard deviation (SD) age was 41 (± 15.02) years, and 40% were aged 31–50 years. With regard to education, 29.5% had a high school education, and 1.9% were illiterate. For employment, 20.5% were manual or unskilled laborers, and 22.9% were dependent or unemployed. Overall, 12.4% were new cases of MDR-TB, and 87.6% were previously treated cases. Of all patients, 33.8% had a history of contact with an MDR-TB patient, 19% had diabetes mellitus, and 10% had HIV. The mean (± SD) distance to the regional TB center was 10.12 (± 6.61) kilometers, and 43.3% lived more than 20 km away. Mean (± SD) travel duration to the regional TB center was 90.10 (± 49.99) minutes, and 24.3% lived more than 120 min away. Of all patients, 61% had high knowledge, 61.9% had good awareness, and 56.7% had a low stigma level.

**Table 1:** Characteristic of MDR-TB patients (*n* = 210)

Variables	Frequency (%)
Demographic factors	
Gender	
Male	131 (62.4)
Female	79 (37.6)
Age	
≤ 30 years	62 (29.5)
31–50 years	84 (40.0)
> 50 years	64 (30.5)
*Mean ± SD (41 ± 15.02), Minimum 18, Maximum 78*	
Family member	
< 4	88 (41.9)
≥ 4	122 (58.1)
*Mean ± SD (4 ± 2.15), Minimum 1, Maximum 12*	
Socio economic factors	
Education	
Illiterate	4 (1.9)
Read and Write	19 (9.0)
Primary school education level	45 (21.4)
Middle school education level	49 (23.3)
High school education level	62 (29.5)
Graduate and above	31 (14.8)
Occupation	
Dependent	48 (22.9)
Manual or unskilled laborer	43 (20.5)
Owned business or self-employee	41 (19.5)
Government staff	34 (16.2)
Private employee	44 (21.0)
Family income	
≤ 200,000 Kyats	56 (26.7)
200,001–400,000 Kyats	125 (59.5)
> 400,000 Kyats	29 (13.8)
*Mean ± SD (307,524.81 ± 121,484.29), Minimum 80,000 Kyats, Maximum 1,000,000 Kyats*	
Disease factors	
Type of patients	
New Cases	26 (12.4)
Previously Treated Cases	184 (87.6)
Relapse	88 (41.9)
Category I Failure	74 (35.2)
Category II Failure	15 (7.1)
Defaulted	7 (3.3)
Contact with MDR-TB patient	
Present	71 (33.8)
Absent	139 (66.2)
Diabetes Mellitus	
Present	40 (19.0)
Absent	170 (81.0)
HIV status	
Present	21 (10.0)
Absent	189 (90.0)
First visited medical provider	
Self-initiative	18 (8.6)
General Practitioner	170 (81.0)
Private Hospital	9 (4.3)
Government Hospital	8 (3.8)
NGOs (PSI)	5 (2.4)
Geographic accessibility	
Residence	
Rural	50 (23.8)
Urban	160 (76.2)
Distance (km)	
> 20 km	91 (43.3)
11–20 km	90 (42.9)
≤ 10 km	29 (13.8)
*Mean ± SD (18.12 ± 6.61), Minimum 8.68, Maximum 31*	
Duration or time spent from residence to TB Center (min)	
> 120 min	51 (24.3)
61–120 min	66 (31.4)
≤ 60 min	93 (44.3)
*Mean ± SD (90.10 ± 49.99), Minimum 30, Maximum 200*	
Consciousness on MDR-TB	
Knowledge Level	
Low Knowledge (< Mean sore)	82 (39.0)
High Knowledge (≥ Mean score)	128 (61.0)
*Mean ± SD (26.27 ± 2.33), Minimum 19, Maximum 33*	
Awareness Level	
Poor Awareness (< Mean sore)	80 (38.1)
Good Awareness (≥ Mean score)	130 (61.9)
*Mean ± SD (56.27 ± 3.12), Minimum 48, Maximum 65*	
Psychological factor	
Stigma Level	
Low Stigma (< Mean sore)	119 (56.7)
High Stigma (≥ Mean score)	91 (43.3)
*Mean ± SD (10.07 ± 4.71), Minimum 3, Maximum 22*	

### Diagnosis and treatment initiation delays

**Fig. 3:**
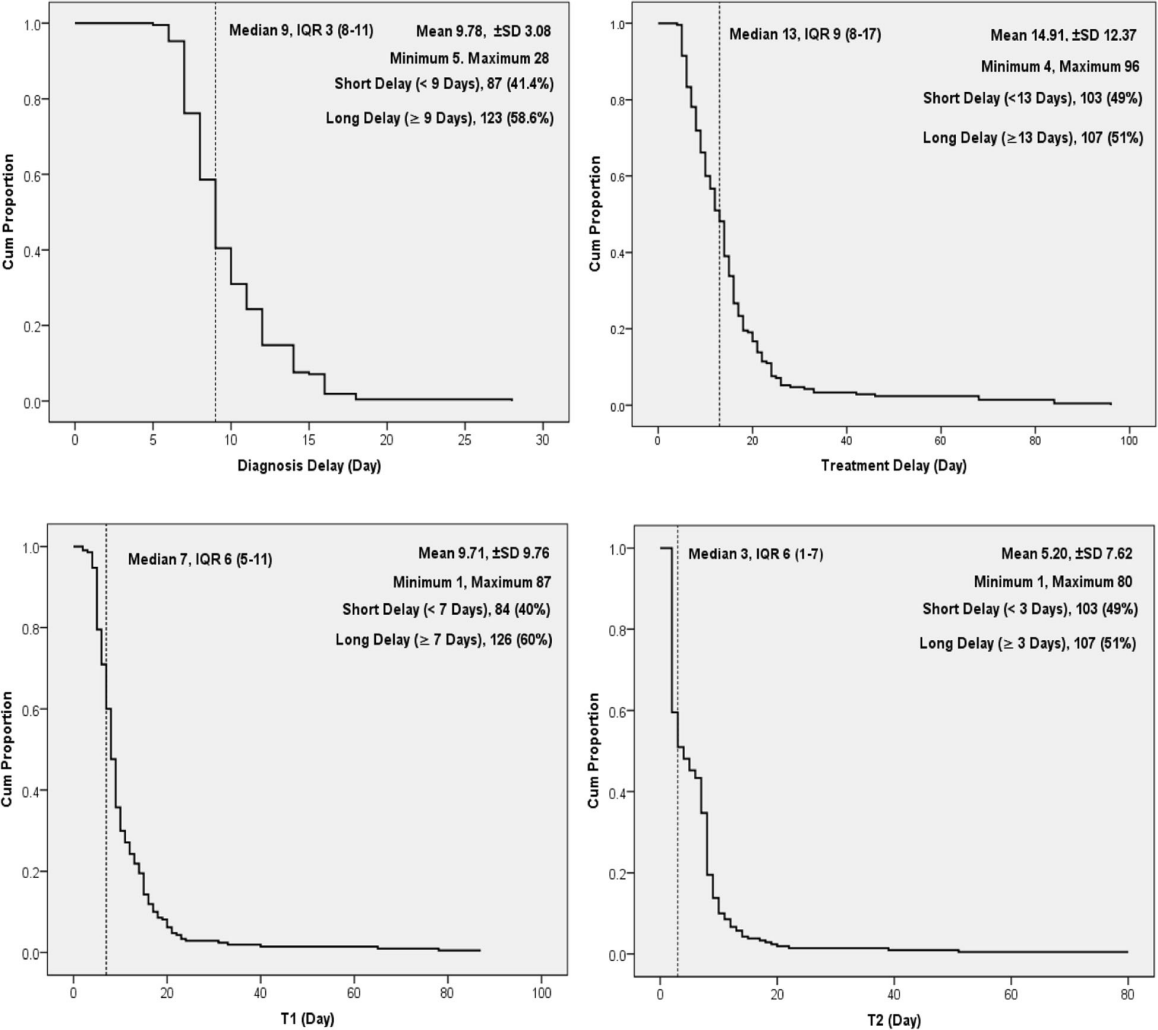
Delay in diagnosis and treatment initiation

As shown in Fig. 3, the median diagnosis delay was 9 days, IQR: 3 (8–11), with a range of 5 to 28 days, and 58.6% experienced long diagnosis delays. The median treatment delay was 13 days, IQR: 9 (8–17), with a range of 4 to 96 days, and 51% experienced long treatment delays. The median T1 delay was 7 days, IQR: 6 (5–11), with a range of 1 to 87 days, and 60% experienced long T1 delays. The median T2 delay was 3 days, IQR: 6 (1–7), with a range of 1 to 80 days, and 51% experienced long T2 delays.

### Factors associated with diagnosis and treatment delays

The factors associated with diagnosis delay were shown in (Table 2). The patients who had less than a middle school education level (AOR = 2.75, 95% CI = 1.22–6.21), those who were working in non-permanent salaried employment (AOR = 3.03, 95% CI = 1.32–6.95), co-existing diabetes mellitus patients (AOR = 5.06, 95% CI = 1.97–13.01), and those who had poor awareness (AOR = 2.99, 95% CI = 1.29–6.92) were significantly associated with long diagnosis delays.

**Table 2:** Factors associated with diagnosis delay among MDR-TB patients (*n* = 210)

Variables	Diagnosis Delay *n* (%)		Univariate analysis		Multivariate analysis	
	< 9 Days	≥ 9 Days	*p* value	COR (95% CI)	*p* value	AOR (95% CI)
Demographic factors						
Sex						
Male	54 (41.2)	77 (58.8)		1.00		1.00
Female	33 (41.8)	46 (58.2)	0.94	0.98 (0.56–1.72)	0.51	0.76 (0.34–1.70)
Age						
≤ 30 years	28 (45.2)	34 (54.8)		1.00		1.00
31–50 years	33 (39.3)	51 (60.7)	0.48	1.27 (0.66–2.47)	0.72	0.86 (0.38–1.95)
> 50 years	26 (40.6)	38 (59.4)	0.61	1.20 (0.59–2.44)	0.09	0.42 (0.15–1.14)
Family member						
≥ 4	52 (42.6)	70 (57.4)		1.00		1.00
< 4	35 (39.8)	53 (60.2)	0.68	1.13 (0.64–1.97)	0.88	0.95 (0.44–2.02)
Socio economic factors						
Education ^a^						
Middle school education level and above	69 (48.6)	73 (51.4)		1.00		1.00
Below middle school education level	18 (26.5)	50 (73.5)	0.003	2.63 (1.39–4.94)	0.02	2.75 (1.22–6.21)
Occupation ^b^						
Employed (permanent salary)	42 (53.8)	36 (46.2)		1.00		1.00
Employed (non-permanent salary)	24 (28.6)	60 (71.4)	0.001	2.92 (1.52–5.59)	0.01	3.03 (1.32–6.95)
Unemployed	21 (43.8)	27 (56.3)	0.27	1.50 (0.73–3.09)	0.28	1.87 (0.59–5.85)
Family income						
> 400,000 Kyats	12 (41.4)	17 (58.6)		1.00		1.00
200,001–400,000 Kyats	52 (41.6)	73 (58.4)	0.98	0.99 (0.44–2.25)	0.44	0.68 (0.26–1.82)
≤ 200,000 Kyats	23 (41.1)	33 (58.9)	0.97	1.01 (0.41–2.52)	0.43	0.63 (0.19–2.01)
Disease factors						
Types of MDR-TB patients						
New	11 (42.3)	15 (57.7)		1.00		1.00
Previously Treated	76 (41.3)	108 (58.7)	0.92	1.04 (0.45–2.39)	0.99	0.99 (0.30–3.29)
Contact with MDR-TB patient						
No	57 (41.0)	82 (59.0)		1.00		1.00
Yes	30 (42.3)	41 (57.7)	0.86	0.95 (0.53–1.69)	0.95	1.02 (0.45–2.31)
Diabetes Mellitus						
Absent	79 (46.5)	91 (53.5)		1.00		1.00
Present	8 (20.0)	32 (80.0)	0.003	3.47 (1.51–7.97)	0.001	5.06 (1.97–13.01)
HIV status						
Absent	80 (42.3)	109 (57.7)		1.00		1.00
Present	7 (33.3)	14 (66.7)	0.43	1.47 (0.57–3.80)	0.16	2.23 (0.74–6.74)
First visit medical provider ^c^						
Self-initiative	12 (66.7)	6 (33.3)		1.00		1.00
General Practitioner	67 (39.4)	103 (60.6)	0.03	3.08 (1.10–8.59)	0.18	2.18 (0.69–6.90)
Hospitals and NGOs	8 (36.4)	14 (63.6)	0.61	3.50 (0.95–12.97)	0.36	2.02 (0.45–9.01)
Geographic accessibility						
Distance (km)						
≤ 10 km	13 (44.8)	16 (55.2)		1.00		1.00
11–20 km	33 (36.7)	57 (63.3)	0.43	1.40 (0.60–3.28)	0.87	1.09 (0.39–3.02)
> 20 km	41 (45.1)	50 (54.9)	0.98	0.99 (0.43–2.29)	0.42	0.55 (0.13–2.29)
Duration or time spent from residence to TB Center (min)						
≤ 60 min	35 (37.6)	58 (62.4)		1.00		1.00
61–120 min	29 (43.9)	37 (56.1)	0.42	0.77 (0.41–1.46)	0.97	0.99 (0.39–2.46)
> 120 min	23 (45.1)	28 (54.9)	0.38	0.74 (0.37–1.47)	0.65	0.75 (0.22–2.61)
Consciousness on MDR-TB						
Knowledge Level						
High Knowledge	56 (43.8)	72 (56.3)		1.00		1.00
Low Knowledge	31 (37.8)	51 (62.2)	0.39	1.28 (0.73–2.26)	0.30	1.54 (0.68–3.52)
Awareness Level						
Good Awareness	61 (46.9)	69 (53.1)		1.00		1.00
Poor Awareness	26 (32.5)	54 (67.5)	0.04	1.84 (1.03–3.28)	0.01	2.99 (1.29–6.92)
Psychological factor						
Stigma Level						
Low Stigma	51 (42.9)	68 (57.1)		1.00		1.00
High Stigma	36 (39.6)	55 (60.4)	0.63	1.15 (0.66–1.99)	0.12	0.50 (0.21–1.20)

^a^Education - categorized as middle school education level and above (middle school education, high school education, and graduate and above) and below middle school education level (illiterate, read and write, and primary school education)

^b^Occupation - categorized as permanent salary employed (government staff and private employee), non-permanent salary employed (dependent, self-employee and unskilled laborer)

^c^First visit medical provider – categorized as self-initiative, general practitioner and, hospitals and NGOs (private hospitals, government hospitals and NGOs)

In (Table 3), as the factors associated with delay in treatment initiation, the patients who were in age of 31–50 years (AOR = 4.50, 95% CI = 1.47–13.97), older than 50 years (AOR = 9.40, 95% CI = 2.55–34.83), those who had a history of contact with MDR-TB patient (AOR = 3.16, 95% CI = 1.29–7.69), those who were living more than 20 km away from the Regional TB Center (AOR = 14.33, 95% CI = 1.91–107.64), and those who had poor awareness (AOR = 4.62, 95% CI = 1.56–13.67) were significantly associated with long treatment delay.

**Table 3:** Factors associated with treatment delay among MDR-TB patients (*n* = 210)

Variables	Treatment Delay *n* (%)		Univariate analysis		Multivariate analysis	
	< 13 Days	≥ 13 Days	*p* value	COR (95% CI)	*p* value	AOR (95% CI)
Demographic factors						
Sex						
Male	74 (56.5)	57 (43.5)		1.00		1.00
Female	29 (36.7)	50 (63.3)	0.006	2.24 (1.26–3.97)	0.19	1.97 (0.70–5.56)
Age						
≤ 30 years	48 (77.4)	14 (22.6)		1.00		1.00
31–50 years	43 (51.2)	41 (48.8)	0.002	3.27 (1.57–6.81)	0.009	4.50 (1.47–13.97)
> 50 years	12 (18.8)	52 (81.3)	0.001	14.86 (6.26–35.29)	0.001	9.40 (2.55–34.83)
Family member						
≥ 4	71 (58.2)	51 (41.8)		1.00		1.00
< 4	32 (36.4)	56 (63.6)	0.002	2.44 (1.39–4.28)	0.57	2.60 (0.97–6.98)
Socio economic factors						
Education ^a^						
Middle school education level and above	82 (57.7)	60 (42.3)		1.00		1.00
Below middle school education level	21 (30.9)	47 (69.1)	0.001	3.06 (1.66–5.65)	0.54	1.37 (0.49–3.78)
Occupation ^b^						
Employed (permanent salary)	56 (71.8)	22 (28.2)		1.00		1.00
Employed (non-permanent salary)	35 (41.7)	49 (58.3)	0.001	3.56 (1.85–6.87)	0.35	1.53 (0.59–4.26)
Unemployed	12 (25.0)	36 (75.0)	0.001	7.64 (3.37–17.31)	0.10	3.25 (0.79–13.39)
Family income						
> 400,000 Kyats	15 (51.7)	14 (48.3)		1.00		1.00
200,001–400,000 Kyats	53 (42.4)	72 (57.6)	0.36	1.46 (0.65–3.27)	0.95	1.04 (0.31–3.50)
≤ 200,000 Kyats	35 (62.5)	21 (37.5)	0.34	0.64 (0.26–1.59)	0.05	0.23 (0.51–1.00)
Disease factors						
Types of MDRTB patient						
New	0 (0.0)	26 (100.0)		–		–
Previously Treated	103 (56.0)	81 (44.0)		–		–
Contact with MDR-TB patient						
No	79 (56.8)	60 (43.2)		1.00		1.00
Yes	24 (33.8)	47 (66.2)	0.002	2.58 (1.42–4.68)	0.01	3.16 (1.29–7.69)
Diabetes Mellitus						
Absent	85 (50.0)	85 (50.0)		1.00		1.00
Present	18 (45.0)	22 (55.0)	0.57	1.22 (0.61–2.44)	0.46	0.67 (0.23–1.97)
HIV status						
Absent	94 (49.7)	95 (50.3)		1.00		1.00
Present	9 (42.9)	12 (57.1)	0.55	1.32 (0.53–3.28)	0.38	1.76 (0.50–6.17)
First visit medical provider^c^						
Self-initiative	13 (72.2)	5 (27.8)		1.00		1.00
General Practitioner	76 (44.7)	94 (55.3)	0.03	3.22 (1.09–9.42)	0.84	1.17 (0.26–5.18)
Hospitals and NGOs	14 (63.6)	8 (36.4)	0.56	1.49 (0.39–5.72)	0.56	0.54 (0.07–4.25)
Geographic accessibility						
Distance (km)						
≤ 10 km	26 (89.7)	3 (10.3)		1.00		1.00
11–20 km	59 (65.6)	31 (34.4)	0.02	4.55 (1.28–16.24)	0.06	5.11 (0.95–27.44)
> 20 km	18 (19.8)	73 (80.2)	0.001	35.15 (9.56–129.18)	0.01	14.33 (1.91–107.64)
Duration or time spent from residence to TB Center (min)						
≤ 60 min	70 (75.3)	23 (24.7)		1.00		1.00
61–120 min	23 (34.8)	43 (65.2)	0.001	5.69 (2.85–11.36)	0.14	2.33 (0.77–7.10)
> 120 min	10 (19.6)	41 (80.4)	0.001	12.48 (5.41–28.80)	0.29	2.38 (0.47–12.01)
Consciousness on MDR-TB						
Knowledge Level						
High Knowledge	79 (61.7)	49 (38.3)		1.00		1.00
Low Knowledge	24 (29.3)	58 (70.7)	0.001	3.89 (2.15–7.06)	0.76	1.17 (0.43–3.16)
Awareness Level						
Good Awareness	80 (61.5)	50 (38.5)		1.00		1.00
Poor Awareness	23 (28.8)	57 (71.3)	0.001	3.97 (2.18–7.22)	0.006	4.62 (1.56–13.67)
Psychological factor						
Stigma Level						
Low Stigma	70 (58.8)	49 (41.2)		1.00		1.00
High Stigma	33 (36.3)	58 (63.7)	0.001	2.51 (1.43–4.41)	0.89	0.93 (0.33–2.66)

^a^Education, ^b^Occupation, ^c^First visit medical provider – same as Table 2

## Discussion

In countries with a high MDR-TB burden, delays in the MDR-TB treatment process remain a challenge for reaching the Sustainable Development Goals targets [29]. The 9-day median diagnosis delay found in this study was longer than the 5-day delay found in a study in Bangladesh [21] but shorter than the 66-day delay found in China [26]. About 59% of the patients in the present study experienced long diagnosis delays (≥ 9 days), compared with 81% experiencing diagnosis delays longer than 90 days in China [26]. These differences in diagnosis delays may have resulted from the use of different diagnosis methods and algorithms.

The 13-day median treatment delay found in this study was longer than the median treatment delays of 5 to 10 days found in previous studies in China [26] and Bangladesh [21, 30]. However, the present finding was shorter than the median treatment delay reported by a study in Taiwan (120 days), which defined treatment delay differently—as the period between sputum collection and the start of treatment [31]. In the present study, 51% of patients experienced long treatment delays (≥ 13 days), compared with 37% in a study in China. Treatment algorithms and the centralization of treatment initiation by Regional TB Centre might influence delay in treatment initiation.

Poor collaboration among medical providers and delays in notifying patients might contribute to unnecessary diagnosis and treatment initiation delays [32]. For the period starting from the date of MDR-TB confirmation to the date of registration at Regional TB Center, T1, the delay might be caused by weaknesses in the notification process by which health providers communicate with patients confirmed to have MDR-TB or in the process through which patients were referred to the Regional TB Center. The communication of results between health facilities and laboratories was not streamlined and resulting long delay between date of MDR-TB confirmation and registration at the Regional TB Center. For the period starting from the date of registration to the date of treatment initiation at Regional TB Center, T2, baseline investigations were conducted at the time of registration, before treatment initiation; it thus could be suggested that the median T2 delay in present study was reasonable.

Patients aged ≥30 years were likely to experience long treatment delay, but this was not associated with diagnosis delay. This may be explained by patients of working age (31–50 years) being more concerned about their employment because of financial hardship. It was also possible that elderly patients (> 50 years) might have some difficulties accessing treatment centers because they generally rely on their family members. Most elderly patients could not come to treatment center alone when their family members were not available for coming together with them. Social supports and pre-treatment counseling both elderly patients and their family members were needed to expand by outreach activity in collaboration with partners. Nevertheless, a previous study found no association between age and treatment delay [26].

Patients with non-permanent salaried employment might be concerned with their employment and face socioeconomic burdens during treatment. Some patients were the primary earners in the family, potentially making it difficult for them to leave work for coming to the diagnosis center. It should be consistency in the provision of the standardized support package including financial support, nutritional support and personal infection control training and masks. On the other hand, patients also might use a lack of time as an excuse to delay treatment initiation because of fear social discrimination [33]. A previous study documented long diagnosis delays among the patients visiting private practitioners for their first consultation [21].

Patients with MDR-TB who also had diabetes mellitus experienced long diagnosis delays; it may be that the double burden of these diseases and poor awareness about TB symptoms hindered these patients’ access to diagnosis centers. Treatment adherence counselling for diabetes patients before referral to diagnostic center should be established by health providers. However, this finding is not in accordance with a study in China study that reported treatment delays to be more common in chronic cases [26]. Concerning long treatment duration, side effects of drugs and misconceptions about disease transmission also made it difficult for MDR-TB contact patients to come to the Regional TB Center for treatment initiation. Systematized contact tracing, active TB screening and monitoring all household contacts during treatment should be integrated into the routine PMDT services [34]. Further, transport reimbursements should be provided for household members as well as community volunteers who were conducting household-level screening.

Awareness about MDR-TB was found to be crucial for minimizing both of delays in diagnosis and treatment [35], and patients with poor awareness might not well essence for early disease confirmation, making it difficult to access diagnosis and treatment centers. Patients with low levels of knowledge and awareness might not seek effective health services early, leading to transmission to the community [33]. Comprehensive health education, pre-test counseling, pre-treatment counseling, treatment adherence counseling and provision of emotional support should be strengthened. Similar to a previous study in Bangladesh [21], the patients in the present study with less than a middle school education were likely to experience long diagnosis delay. Hence, comprehensive health education should be more provided to all patients with MDR-TB and the community, highlighted on patients with low education level, to improve their awareness on MDR-TB.

In addition to financial burdens, previous anti-TB treatments, chronic diseases, initial hospitalization, and waiting list for treatment to be associated with treatment delays [21, 26, 33]. One reason was that these variables were associated with difficulty accessing medical facilities because of geographical barriers, which, in turn, could affect disease progression and transmission in the community [33].

The present study highlighted that the MDR-TB treatment network would be enhanced by the expansion of decentralized MDR-TB treatment centers to provide diagnostic capacities with Xpert MTB/RIF, treatment initiation, baseline lab investigation, and adverse effects of second line drugs management. The study also supported recommendations of previous studies to establish more treatment centers to reduce patients’ financial burdens and treatment delays [36, 37]. To achieve universal accessibility, the diagnostic network of Xpert MTB/RIF machines should be expanded to cover hard-to-reach areas. Continuous expansion of proper sample transportation system and health information amongst health facilities would also facilitate nationwide coverage. Myanmar is moving toward a streamlined diagnostic network with intensified focus on improving access to quality health care services and patient-centered care.

### Limitations of the study

There might be other factors influencing delays that were not included in this study; for example, the perceptions of MDR-TB participants regarding drug resistant TB case management. Further qualitative studies might reveal hidden reasons for delay in diagnosis and treatment. With the nature of the study as cross-sectional survey, treatment outcomes of patients could not be traced; conducting further prospective cohort studies would be useful to find out the effect of delays on treatment outcomes among MDR-TB patients. Results of this study might be generalized to elsewhere in which the MDR-TB patients were treated and cared in similar settings to Yangon Regional TB Center. However, if the case management process and infrastructure of the TB healthcare services are different (even in other Regional/State TB Centers in Myanmar), the study results might varied, particularly among those with greater diversity demographic, socioeconomic, diseases factors and geographical healthcare accessibility.

## Conclusions

In Myanmar, long diagnosis and treatment delays were still occurred in high proportion of MDR-TB patients after installation and providing of rapid diagnosis test. It could be improved by strengthening in comprehensive health education, enhancing treatment adherence counseling to patients. On the healthcare provider side, providing diagnostic facilities with availability of Xpert MTB/RIF, DST and RR screening, expanding number of decentralized MDR-TB treatment centers all district level and townships, strengthening health information between diagnosis and treatment centers, ensuring consistency in a standardized support package, implementing adequate staffing by improving human resources, and conducting systematized contact tracing and active TB screening would be effective ways to minimize diagnosis and treatment delays.

## Abbreviations

AOR: Adjusted odds ratio
CI: Confidence interval
COR: Crude odds ratio
DOTS: Directly observed treatment short-course
DST: Drug susceptibility testing
HIV: Human immunodeficiency virus
IQR: Interquartile ranges
MDR-TB: Multidrug-resistant tuberculosis
NTP: National Tuberculosis Programme
PMDT: Programmatic management of drug-resistant tuberculosis
RR: Rifampicin resistance
SD: Standard deviation
SPSS: Statistical package for the social sciences
TB: Tuberculosis
WHO: World Health Organization
